# Independent and Combined Associations of Urinary Heavy Metal Exposures with Serum α-Klotho in Middle-Aged and Older Adults

**DOI:** 10.3390/toxics13040237

**Published:** 2025-03-24

**Authors:** Xinliang Zheng, Wenxin Zhou, Zhuoying Jiang, Chan Ding, Minqian Feng, Yongxin Li, Fitri Kurniasari, Shuanghua Xie, Huadong Xu

**Affiliations:** 1 School of Public Health, Zhejiang Provincial People’s Hospital (Affiliated People’s Hospital), Hangzhou Medical College, 182 Tianmushan Road, Xihu District, Hangzhou 310013, China; zxl2695291160@163.com (X.Z.); zzzhouwx@foxmail.com (W.Z.); jiangzhuoying0304@163.com (Z.J.); 130232023252@hmc.edu.cn (C.D.); 130232024250@hmc.edu.cn (M.F.); 21617018@zju.edu.cn (Y.L.); 2 Department of Environmental Health, Faculty of Public Health, University of Indonesia, Depok 16424, West Java, Indonesia; fitri.kurniasari04@ui.ac.id; 3 Department of Central Laboratory, Beijing Obstetrics and Gynecology Hospital, Capital Medical University, Beijing Maternal and Child Health Care Hospital, Beijing 100026, China

**Keywords:** α-Klotho, urinary metals, NHANES, metal mixtures, aging biomarkers

## Abstract

α-Klotho is an anti-aging protein linked to various age-related diseases. Environmental metal exposure has been associated with oxidative stress and aging, but its effect on α-Klotho levels remains unclear. This study investigated the relationship between urinary metal concentrations and serum α-Klotho levels using data from the National Health and Nutrition Examination Survey (NHANES) 2007–2016 cycles. A total of 4071 adults aged 40 to 79 years were included in the analysis. After adjusting for potential confounders, positive associations were found between serum α-Klotho levels and barium (Ba), cesium (Cs), and molybdenum (Mo), while tungsten (W) and uranium (U) were negatively correlated with α-Klotho levels. The combined effects of multiple metals were further analyzed using the qgcomp model, which demonstrated a negative correlation between increased metal mixtures and serum α-Klotho levels. Specifically, U, total arsenic (t-As), W, cadmium (Cd), antimony (Sb), and lead (Pb) contributed to the reduction of α-Klotho levels, while Ba, Cs, dimethylarsinic acid (DMA), Mo, thallium (Tl), and cobalt (Co) were positively associated with α-Klotho levels. These findings suggest that exposure to certain metals, particularly in combination, may reduce serum α-Klotho levels, potentially accelerating aging processes. Further studies should investigate the underlying mechanisms responsible for these associations.

## 1. Introduction

The Klotho protein family, including α-Klotho and β-Klotho, plays a central role in regulating aging and longevity. Of particular interest is α-Klotho, a soluble form of the protein that has been shown to exert protective effects on various organ systems, such as the cardiovascular, renal, and nervous systems [1,2]. Animal studies have demonstrated that α-Klotho contributes to an extended lifespan and delayed onset of age-related diseases, such as kidney disease and neurodegeneration [1,3,4,5]. In humans, higher α-Klotho levels have been linked to reduced all-cause mortality, highlighting its potential as a therapeutic target for age-related conditions [6]. Recent research has further linked α-Klotho to the pathophysiology of chronic diseases, such as cardiovascular disease [7], cancer [8], and diabetes [9], highlighting its potential as both a biomarker for aging and a therapeutic target for various age-related diseases.

Environmental exposure to heavy metals is a widespread global health concern. These metals can enter the human body through inhalation, ingestion, and dermal absorption, leading to various adverse health outcomes, including neurodevelopmental disorders, cardiovascular diseases, and cancer [10]. Arsenic (As), lead (Pb), and cadmium (Cd) are well-documented toxic metals [11,12], while antimony (Sb), tungsten (W), and molybdenum (Mo) also pose potential health risks. Sb exposure has been associated with oxidative stress and immunotoxicity [13], W has been implicated in carcinogenesis and systemic toxicity [14], and Mo can be toxic at high levels, impairing enzymatic functions and metabolism [15]. Environmental metal exposure has also been linked to accelerated aging processes, indicating that metals contribute to increased oxidative stress, DNA damage, and impaired cellular function, all of which are hallmarks of aging [16]. Therefore, examining the relationship between metal exposure and aging biomarkers, such as α-Klotho, is critical for understanding how environmental pollutants influence aging and contribute to age-related diseases.

A growing body of research has explored the relationship between α-Klotho levels and environmental pollutants, including organic contaminants such as polycyclic aromatic hydrocarbons [17], perchlorates, nitrates, thiocyanates [18], and dichlorobenzene [19]. Emerging studies have elucidated the impact of heavy metal exposure on α-Klotho levels. A study using NHANES data found that higher Pb levels are associated with lower serum α-Klotho, further supporting the link between heavy metal exposure and aging-related processes [20]. Additionally, exposure to Cd and Pb has been linked to oxidative stress, with evidence indicating that α-Klotho homeostasis may be disrupted in individuals with kidney dysfunction, especially at more severe stages [21]. Another investigation demonstrated that α-Klotho partially mediated the association between blood Pb levels and estimated glomerular filtration rate (eGFR), suggesting a potential role of α-Klotho in Pb-induced renal dysfunction [22]. Furthermore, studies on three essential elements identified a negative correlation between serum α-Klotho and blood copper (Cu) levels [23]. These findings underscore the importance of investigating the effects of heavy metal exposure on serum α-Klotho levels, especially the potential synergistic effects of multiple metal exposures on health. Given the ubiquity and persistence of metal pollutants, it is essential to explore how combined metal exposure influences α-Klotho levels, particularly in the general population.

The goal of this study was to investigate the association between urinary metal concentrations and serum α-Klotho levels using data from the National Health and Nutrition Examination Survey (NHANES), which is a nationally representative survey that provides comprehensive health data on the U.S. population, making it an invaluable resource for environmental health studies [24,25]. We used multiple linear regression and quantile g-computation (qgcomp) models to evaluate the independent and combined effects of urinary metals on serum α-Klotho levels. By examining a wide range of urinary metals and their mixtures, this study aims to provide new insights into the relationship between environmental metal exposure and aging biomarkers.

## 2. Materials and Methods

### 2.1. Study Design and Participants

**Figure 1:**
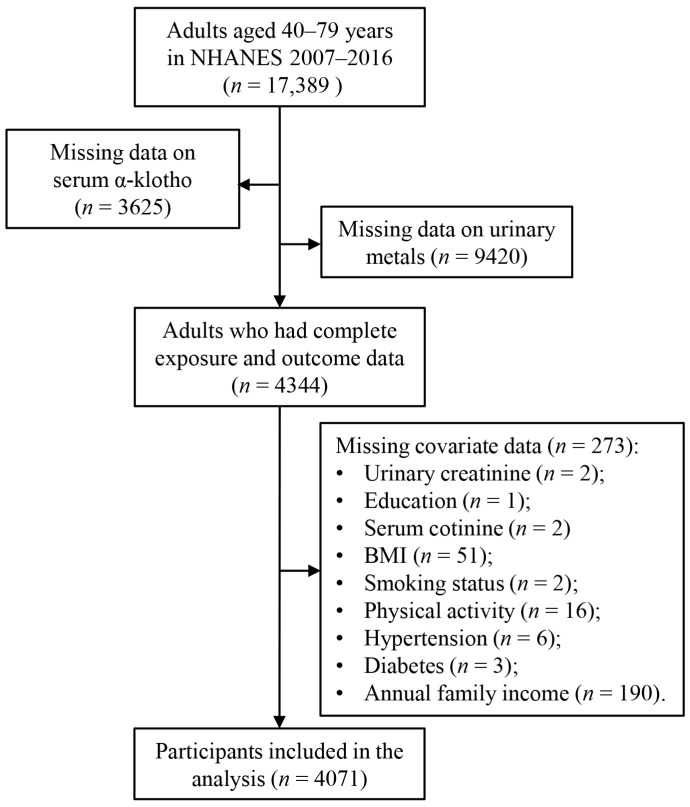
Flow diagram of selection of study participants from NHANES 2007–2016.

This study analyzed data from five cycles of the NHANES conducted between 2007 and 2016 (2007–2008, 2009–2010, 2011–2012, 2013–2014, 2015–2016). NHANES emphasizes environmental exposures and their impacts on health outcomes. Participants were selected based on the availability of data on urinary metals and serum α-Klotho levels, as shown in Figure 1. Initially, 17,389 adults aged 40–79 years were included in the analysis, as serum α-Klotho levels were only assessed in individuals within this age group. After excluding participants with incomplete data on serum α-Klotho (*n* = 3625), urinary metals (*n* = 9420), or covariates (*n* = 273), the final study population consisted of 4071 participants. The NHANES study protocol was reviewed and approved by the Institutional Review Board of the National Center for Health Statistics (NCHS), and informed consent was obtained from all participants. No additional ethical approval was required for this secondary data analysis.

### 2.2. Measurement of Urinary Metal Levels

Urine samples were collected, stored at −30 °C, and subsequently shipped to the National Center for Environmental Health (NCEH) for analysis. Urinary concentrations of total arsenic (t-As), barium (Ba), cobalt (Co), Mo, cesium (Cs), Cd, Pb, Sb, thallium (Tl), W, and uranium (U) were quantified using inductively coupled plasma mass spectrometry (ICP-MS). For As species, including dimethylarsinic acid (DMA), arsenobetaine, monomethylarsonic acid, arsenocholine, arsenous (III) acid, and arsenic (V) acid, high-performance liquid chromatography (HPLC) was used. However, only DMA had a detection rate exceeding 70%, while other arsenic species exhibited lower detection rates. For elements measured by ICP-MS and other techniques, values below the limit of detection (LOD) were imputed as LOD divided by the square root of two, following NHANES protocols. Detailed information on laboratory procedures for measuring urinary metals and arsenic species can be found on the NHANES website.

### 2.3. Measurement of Serum α-Klotho Levels

Serum samples were stored at −80 °C until further analysis. α-Klotho levels were measured using an ELISA kit from IBL International (Gunma, Japan). The assay was validated prior to the study, demonstrating excellent sensitivity (4.33 pg/mL, compared to the manufacturer’s claim of 6.15 pg/mL) and linearity (R^2^ = 0.998 and 0.997 for high and low α-Klotho concentrations). Intra-assay precision was 3.2–3.9% for recombinant samples and 2.3–3.3% for human samples, while inter-assay precision was 2.8–3.5% for recombinant samples and 3.4–3.8% for human samples. All samples were analyzed in duplicate, and results met the laboratory’s acceptance criteria. Additional methodological details can be found on the NHANES website (https://wwwn.cdc.gov/Nchs/Data/Nhanes/Public/2015/DataFiles/SSKL_I.htm) (accessed on 5 December 2024).

### 2.4. Covariates

Several covariates were selected based on their known associations with α-Klotho levels, as reported in previous literature [19,20,26,27], including age, sex, smoking status, physical activity, body mass index (BMI), diabetes mellitus, hypertension, race/ethnicity, education level, annual family income, marital status, serum cotinine, and NHANES cycle. Urinary creatinine levels were included to adjust for variability in urine concentration.

### 2.5. Statistical Analysis

Statistical analyses were performed using R software (version 4.2.1). Group differences in α-Klotho levels were evaluated using the Mann–Whitney U test for two-group comparisons and the Kruskal–Wallis test for multiple-group analyses. Urinary metal concentrations were analyzed both as continuous variables (after natural logarithmic transformation) and in quartiles. Multiple linear regression models were used to evaluate the association between serum α-Klotho levels and urinary metal concentrations, with results presented as the estimated percent changes in serum α-Klotho levels and their corresponding 95% confidence intervals (CIs) according to previous studies [28]. The *p*-value for trend was calculated by fitting urinary metal quartiles into linear models to assess dose–response relationships. All regression models were adjusted for age, sex, race/ethnicity, BMI, annual family income, smoking, education, marital status, hypertension, diabetes, NHANES cycle, physical activity, serum cotinine, and urinary creatinine.

In addition to analyzing single metal effects, the combined effects of multiple urinary metals on serum α-Klotho levels were assessed by adjusting for other metals. Finally, to investigate the impact of metal mixtures, the qgcomp model was implemented in the “qgcomp” R package. This model assigns positive or negative weights to urinary metals, estimating their combined exposure effect. The “qgcomp boot” function was used to assess the linearity of the exposure–response relationship and estimate the overall marginal effect.

## 3. Results

### 3.1. Characteristics of Study Participants and Serum α-Klotho Levels

A total of 4071 adults aged 40 to 79 years were included (Table 1), comprising 1999 males (49.1%) and 2072 females (50.9%). Serum α-Klotho levels varied significantly by age, sex, and race/ethnicity. Participants aged 40–49 years had the highest α-Klotho levels (883.5 ± 338.5 pg/mL), while those aged ≥ 70 years had the lowest levels (796.8 ± 264.3 pg/mL) (*p* < 0.001). Females had higher α-Klotho levels (882.4 ± 340.6 pg/mL) compared to males (819.5 ± 269.5 pg/mL) (*p* < 0.001), and non-Hispanic Black participants had the highest levels (905.5 ± 358.3 pg/mL) (*p* < 0.001). Serum α-Klotho levels were significantly higher in participants with hypertension (861.9 ± 309.9 pg/mL) compared to those without hypertension (839.3 ± 308.3 pg/mL) (*p* = 0.002). Underweight participants also had significantly higher α-Klotho (919.7 ± 331.3 pg/mL) than those in other BMI categories (*p* = 0.002). Significant variations in α-Klotho levels were also observed across NHANES cycles (*p* < 0.001), with the highest levels in the 2011–2012 cycle (897.5 ± 330.9 pg/mL) and the lowest in the 2015–2016 cycle (817.7 ± 329.3 pg/mL). Regarding marital status, participants who were never married had significantly higher serum α-Klotho levels (890.6 ± 405.3 pg/mL) compared to those who were married/cohabiting (849.1 ± 288.1 pg/mL) (*p* = 0.040). However, no significant differences were observed across education levels, family income, serum cotinine levels, physical activity, smoking status, or diabetes status.

### 3.2. Distribution of Urinary Metal Concentrations

The distribution of urinary metal concentrations among the study participants is presented in Table 2. The detection rates for the 12 urinary metals ranged from 70.4% (for Sb) to 100% (for Cs and Mo). The highest mean urinary concentrations were observed for Mo at 51.28 μg/L, followed by t-As at 19.86 μg/L and DMA at 5.72 μg/L. The lowest mean concentrations were found for uranium (U) at 0.0121 μg/L and Sb at 0.074 μg/L. The 95th percentile concentrations ranged from 0.0351 μg/L for U to 139.40 μg/L for Mo, indicating considerable variability in exposure levels across participants. Additionally, positive correlations were observed among the urinary metal concentrations, as shown in Figure S1, suggesting that participants with higher levels of one metal often had elevated levels of others. These inter-metal correlations highlighted the complexity of metal exposures in this population.

### 3.3. Relationships Between Urinary Metals and Serum α-Klotho Levels

The relationships between individual urinary metal concentrations and serum α-Klotho levels were assessed after adjusting for potential confounders (Table S1). After adjusting for age, sex, race/ethnicity, BMI, annual family income, education, marital status, hypertension, diabetes, NHANES cycle, physical activity, serum cotinine, and urinary creatinine, significant positive associations were observed for Ba (percent change = 1.96, 95% CI: 1.19–2.74), Cs (percent change = 2.67, 95% CI: 1.21–4.14), and Mo (percent change = 1.05, 95% CI: 0.05–2.05). Quartile-based analyses revealed that participants in higher quartiles (Q2–Q4) of urinary Ba, Q3–Q4 of Cs, and Q4 of Mo exhibited significantly elevated serum α-Klotho levels compared to the reference quartile (Q1). Conversely, inverse associations were identified for W (percent change = −0.90, 95% CI: −1.69 to −0.10) and U (percent change = −1.65, 95% CI: −2.43 to −0.86). Participants in Q4 of W and Q3–Q4 of U demonstrated significantly lower serum α-Klotho levels relative to Q1.

Subsequently, using mutually adjusted models, the combined effects of multiple urinary metals on serum α-Klotho levels were examined (Table 3). After adjusting for other covariates and metals, Ba, Cs, and Mo remained positively associated with serum α-Klotho levels, with percent change values of 2.10 (95% CI: 1.24, 2.96), 2.81 (95% CI: 0.86, 4.80), and 1.40 (95% CI: 0.22, 2.59), respectively. In contrast, W and U were negatively associated with serum α-Klotho levels (W: percent change = −0.97, 95% CI: −1.88, −0.05; U: percent change = −1.58, 95% CI: −2.42, −0.74). Participants with higher urinary levels of Ba and Cs (Q2, Q3, Q4) had significantly higher serum α-Klotho levels, while those with elevated levels of Sb and U (Q2, Q3, Q4) exhibited significantly lower serum α-Klotho levels.

### 3.4. Association Between Metal Mixtures and Serum α-Klotho by the Qgcomp Model

**Figure 2:**
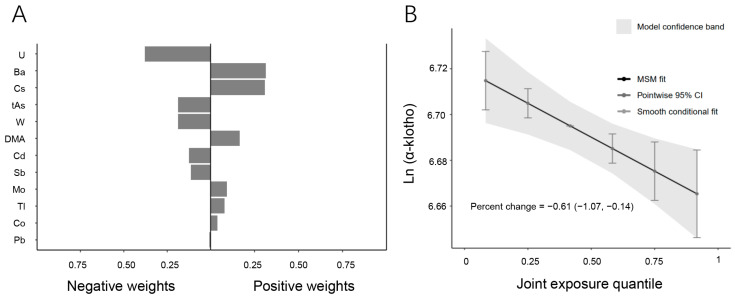
Associations between metal mixtures and serum α-Klotho by the qgcomp model. (**A**) Direction and magnitude of the assigned weights for each metal in association with serum α-Klotho. (**B**) Combined effects of twelve urinary metals levels on serum α-Klotho (95% CI). The model was adjusted for age, sex, race/ethnicity, BMI, annual family income, smoking, education, marital status, hypertension, diabetes, NHANES cycle, physical activity, serum cotinine, and urinary creatinine.

To investigate the combined effects of multiple urinary metals on serum α-Klotho levels, the qgcomp model was employed (Figure 2). The regression index weights revealed that U, t-As, W, Cd, Sb, and Pb contributed negatively to serum α-Klotho levels, with U being the primary contributor to the overall negative association. In contrast, Ba, Cs, DMA, Mo, Tl, and Co showed positive associations with serum α-Klotho levels, with Ba having the greatest positive effect. Furthermore, the combined effect of the metal mixture was analyzed based on the quantile range (Figure 2B). The results demonstrated a negative correlation between the mixture of twelve urinary metals and serum α-Klotho levels. A linear decrease in serum α-Klotho levels was observed across increasing quartiles of the metal mixture, with a percent change of −0.61 (95% CI: −1.07, −0.14) (*p* = 0.002), suggesting that higher exposure to multiple metals is associated with reduced serum α-Klotho levels.

## 4. Discussion

This study comprehensively analyzed the relationship between urinary metal exposures and serum α-Klotho levels using data from the NHANES 2007–2016 cycles [20,29]. While previous studies have explored the associations between α-Klotho and environmental pollutants, especially organic chemicals, the effects of mixed metal exposure on α-Klotho levels remain largely unexplored. Our study is the first to systematically examine both individual and combined metal exposures and their effects on α-Klotho levels in a large, nationally representative sample. This research filled a critical gap in the literature by addressing the previously unexplored relationship between mixed metal exposure and α-Klotho levels.

Although the relationship between metal exposure and α-Klotho remains under investigation, prior studies have demonstrated that heavy metals could impact α-Klotho levels, primarily through oxidative stress and kidney function impairment [10,30]. Previous research has shown that Pb exposure negatively correlates with α-Klotho levels and mediates its effect on kidney function, while Cd and Pb have been linked to oxidative-stress-related Klotho dysregulation, particularly in individuals with renal impairment [21,22]. Additionally, studies on trace elements found that a combined effect of selenium (Se), Cu, and zinc (Zn) decreased α-Klotho concentrations [23]. Our study extends these findings by assessing the effects of multiple metals on α-Klotho simultaneously, rather than focusing on single-metal exposures. We observed that urinary W and U were negatively associated with α-Klotho levels, and our quantile g-computation model revealed a dose-dependent decrease in α-Klotho with increasing metal mixture exposure. These findings suggest that exposure to multiple metals may have a cumulative or synergistic effect, amplifying their negative impact on aging biomarkers. Unlike previous studies that reported significant associations between Cd and Pb with α-Klotho [20], we did not observe statistically significant effects for these metals. This discrepancy may be attributed to differences in study design, population characteristics, or the influence of co-exposures in our multi-metal analysis, which could obscure individual metal effects.

Given the central role of α-Klotho in aging and disease prevention, it is important to consider what constitutes a normal reference range for this biomarker. Currently, there is no universally established reference range for serum α-Klotho protein levels. Most studies on α-Klotho concentrations are based on data from the NHANES [19,20,31,32,33,34]. In our study, the quartile-based analysis of α-Klotho levels provides insight into how varying degrees of metal exposure may affect this anti-aging protein. While the specific health effects of small fluctuations in α-Klotho levels remain unclear, previous research suggests that reduced α-Klotho levels are associated with aging-related diseases. In this context, the observed associations between metal exposure and α-Klotho variations may have long-term implications for health and aging processes.

Our study also compared metal-associated effects on α-Klotho between different demographic groups. Notably, our analysis revealed that individuals who were never married exhibited higher serum α-Klotho levels than those who were married or cohabiting. This observation might reflect differences in psychosocial stress or lifestyle behaviors, which can influence oxidative stress [35] and, in turn, α-Klotho expression. Similarly, individuals with hypertension had significantly higher α-Klotho levels than those without hypertension. Previous studies have suggested that lower α-Klotho levels are associated with an increased risk of hypertension, identifying it as a potential risk factor for elevated blood pressure [4,36]. This discrepancy between our findings and prior research may be due to differences in study populations, measurement methods, or unmeasured confounders. The higher α-Klotho levels observed in hypertensive individuals in our study could represent a compensatory response to vascular stress or endothelial dysfunction. Additionally, potential residual confounding from factors such as medication use, kidney function, or inflammatory status may have influenced the observed association [6,37]. Moreover, α-Klotho levels varied significantly across NHANES cycles. This variation might reflect underlying population-level differences in environmental exposures, dietary patterns, or methodological factors related to sample collection and processing. Future studies should explore whether these variations are linked to temporal trends in metal exposure or other environmental stressors that may impact aging and disease susceptibility.

Interestingly, while most non-essential metals were associated with lower α-Klotho levels, we found that Ba and Mo were positively correlated with α-Klotho. Both metals are considered essential trace elements with antioxidative properties that may counteract oxidative-stress-induced α-Klotho depletion [15,38]. These metals may support the maintenance of α-Klotho levels, counteracting the oxidative damage caused by other environmental stressors. Furthermore, the potential therapeutic implications of these metals in maintaining α-Klotho levels could warrant further investigation into their possible use in anti-aging interventions. This contrasts with the negative effects observed for heavy metals, underscoring the complexity of metal interactions in biological systems. In addition to these compounding effects, it is plausible that antagonistic interactions may also occur between certain metals, either through functional interference or competition at receptor sites [39], which could modify the overall impact on α-Klotho levels. Further research is needed to determine the specific biological roles of different metals in modulating aging processes.

Moreover, our study emphasized the significance of considering metal mixtures rather than focusing solely on individual metal exposures. Using the quantile qgcomp model, we observed a clear dose–response relationship between increasing quartiles of metal mixtures and decreasing serum α-Klotho levels. This suggested that exposure to multiple metals in combination might have compounded or synergistic effects, amplifying their negative impact on aging biomarkers. This finding highlighted the necessity of studying metal mixtures, as they more accurately reflected real-world environmental exposures [40]. Previous research had predominantly focused on individual metal exposures [20,34], but our study demonstrated that the combined effect of multiple metals was likely stronger and more significant in influencing health outcomes, particularly in the context of aging.

The biological mechanisms through which metals influenced α-Klotho levels remain to be fully understood. However, oxidative stress appeared to be a central pathway. α-Klotho is a potent antioxidant and anti-inflammatory protein, and its deficiency is associated with increased ROS production and exacerbated oxidative stress [32]. Previous studies suggested that metals like Cd and Pb, known for their ability to induce oxidative damage, might reduce α-Klotho production, thereby promoting aging [20]. On the other hand, metals like Ba and Mo, which possess antioxidative properties [41,42], might mitigate oxidative damage and protected against age-related cellular dysfunction. These findings underscore the complexity of metal toxicity, as certain metals might have dual effects depending on exposure levels, the presence of other metals, and individual susceptibility.

Despite the valuable insights provided by our study, several limitations must be acknowledged. First, while urinary metals are widely used as biomarkers of exposure, they do not fully capture the total body burden of metals. Additionally, urinary metal concentrations might not reflect long-term accumulation, particularly for metals that accumulate in tissues over time [43]. Second, due to the nature of our cross-sectional study, establishing cause-and-effect relationships was not feasible. Longitudinal studies are needed to validate these associations and better understand the long-term effects of metal exposure on α-Klotho levels and aging. Furthermore, while we adjusted for several confounders, unmeasured factors, such as genetic predisposition, diet, and lifestyle, might also affect the relationship between metal exposure and α-Klotho levels. Moreover, we primarily examined the effects of individual metals and overall metal mixtures but did not explore specific interactions between chemically similar metals, such as Sb + As or Cd + Pb, which may exert unique combined effects. Future research should employ interaction models or machine learning algorithms to investigate whether certain metal pairs have synergistic or antagonistic effects on α-Klotho levels. Lastly, the potential impact of repeated freezing and thawing cycles on serum α-Klotho measurements cannot be ruled out, which might have introduced some variability in the results.

## 5. Conclusions

This study was the first to investigate the effects of twelve urinary metals on serum α-Klotho levels using the NHANES database. Our findings indicated that exposure to metals, especially in combination, was associated with decreased serum α-Klotho levels, potentially influencing aging processes. The identification of both positive and negative correlations underscored the complex biological effects of metals on aging. Further research is needed to clarify the mechanisms through which metals, individually and in mixtures, regulate α-Klotho and impact health. Longitudinal studies should explore causal relationships, while mechanistic investigations can elucidate metal-induced effects on α-Klotho regulation. Additionally, assessing the combined toxicity or protective effects of metal mixtures and the therapeutic potential of metals positively linked to α-Klotho may provide valuable insights into aging-related interventions. Despite its limitations, this study provided a foundation for future research on environmental metal exposure and its impact on aging biomarkers.

## Figures and Tables

**Table 1:** Distribution of serum α-Klotho levels based on the characteristics of study participants in the NHANES 2007–2016.

Characteristic	N	(%)	Serum α-Klotho Levels (Mean ± SD in pg/mL)	*p* Value
Age, years				<0.001
40–49	1144	28.10	883.5 ± 338.5	
50–59	1059	26.01	870.1 ± 306.9	
60–69	1142	28.05	837.0 ± 302.2	
≥70	726	17.83	796.8 ± 264.3	
Sex				<0.001
Male	1999	49.10	819.5 ± 269.5	
Female	2072	50.90	882.4 ± 340.6	
Race/ethnicity				<0.001
Mexican American	632	15.52	857.8 ± 297.9	
Non-Hispanic White	1775	43.60	817.0 ± 282.6	
Non-Hispanic Black	788	19.36	905.5 ± 358.3	
Others	876	21.52	868.2 ± 313.4	
Education				0.088
<High school	1119	27.49	854.3 ± 337.6	
High school	871	21.40	829.7 ± 275.1	
>High school	2081	51.12	859.1 ± 306.7	
Annual family income				0.970
<USD 25,000	1279	31.42	855.0 ± 323.2	
USD 25,000–64,999	1406	34.54	845.6 ± 293.9	
≥USD 65,000	1386	34.05	854.2 ± 311.6	
Marital status				0.040
Married/cohabiting	2645	64.97	849.1 ± 288.1	
Widowed/divorced/separated	1083	26.60	844.9 ± 323.4	
Never married	343	8.43	890.6 ± 405.3	
Smoking status				<0.001
Never	2081	51.12	872.9 ± 304.6	
Current	768	18.87	833.6 ± 314.8	
Former	1222	30.02	826.4 ± 311.3	
BMI, kg/m^2^				0.002
Underweight (<18.5)	40	0.98	919.7 ± 331.3	
Normal weight (18.5–24.9)	953	23.41	875.9 ± 315.3	
Overweight (25–29.9)	1415	34.76	836.9 ± 294.2	
Obese (≥30)	1663	40.85	848.3 ± 317.0	
Physical activity				0.879
Sedentary	1197	29.40	860.5 ± 331.0	
Insufficient	596	14.64	846.3 ± 300.6	
Moderate	556	13.66	839.9 ± 285.4	
High	1722	42.30	850.8 ± 304.1	
Diabetes				0.123
No	3356	82.44	839.6 ± 326.9	
Yes	715	17.56	854.0 ± 305.4	
Hypertension				0.002
No	2189	53.77	839.3 ± 308.3	
Yes	1882	46.23	861.9 ± 309.9	
Serum cotinine				0.421
<LOD	1184	29.08	854.3 ± 294.3	
≥LOD	2887	70.92	850.4 ± 315.3	
NHANES cycle				<0.001
2007–2008	910	22.35	851.4 ± 330.0	
2009–2010	835	20.51	845.4 ± 290.7	
2011–2012	733	18.01	897.5 ± 330.9	
2013–2014	807	19.82	849.1 ± 254.3	
2015–2016	786	19.31	817.7 ± 329.3	

**Table 2:** The distributions of urinary metals among study participants in the NHANES 2007–2016.

Elements	Detection Rates (%)	Mean (μg/L)	Percentile (μg/L)				
			5th	25th	Median	75th	95th
t-As	99.2	19.86	1.56	3.76	7.66	16.60	63.87
DMA	78.5	5.72	1.20	2.00	3.59	6.38	17.20
Ba	99.2	1.85	0.21	0.53	1.07	2.15	5.40
Cd	96.5	0.441	0.049	0.145	0.281	0.547	1.368
Co	99.7	0.499	0.090	0.202	0.336	0.535	1.271
Cs	100	5.11	1.23	2.70	4.39	6.58	11.11
Mo	100	51.28	7.42	20.10	38.10	66.04	139.40
Pb	98.7	0.71	0.10	0.27	0.48	0.83	1.89
Sb	70.4	0.074	0.016	0.028	0.046	0.078	0.192
Tl	99.5	0.178	0.038	0.087	0.149	0.233	0.407
W	82.9	0.110	0.013	0.027	0.060	0.121	0.336
U	84.1	0.0121	0.0014	0.0028	0.0055	0.0113	0.0351

**Table 3:** Associations of multiple urinary metals with serum α-Klotho levels in the NHANES 2007–2016.

Metals	Percent Change of Serum α-Klotho (95% CI) ^a^					*p* for Trend
	Continuous	Q1	Q2	Q3	Q4	
tAs	−0.62 (−1.66, 0.42)	Ref.	−0.83 (−3.28, 1.68)	−2.40 (−5.14, 0.43)	−2.06 (−5.10, 1.08)	0.159
DMA	−0.35 (−1.92, 1.25)	Ref.	−1.17 (−3.61, 1.33)	0.70 (−2.28, 3.76)	0.21 (−3.20, 3.74)	0.646
Ba	2.10 (1.24, 2.96) ***	Ref.	4.46 (2.36, 6.61) ***	4.32 (2.05, 6.64) ***	5.12 (2.65, 7.65) ***	<0.001
Cd	−0.69 (−1.75, 0.38)	Ref.	−0.55 (−3.22, 2.19)	−0.62 (−2.89, 1.70)	−0.14 (−2.23, 2.00)	0.586
Co	−0.28 (−1.49, 0.95)	Ref.	−0.42 (−2.61, 1.83)	0.28 (−2.26, 2.89)	1.12 (−1.68, 3.99)	0.290
Cs	2.81 (0.86, 4.80) **	Ref.	1.75 (−0.66, 4.21)	4.46 (1.54, 7.47) **	4.32 (1.03, 7.72) *	0.005
Mo	1.40 (0.22, 2.59) *	Ref.	−0.39 (−2.54, 1.82)	0.70 (−1.78, 3.23)	0.35 (−2.42, 3.20)	0.641
Pb	−0.48 (−1.65, 0.70)	Ref.	0.35 (−1.81, 2.55)	−0.48 (−2.90, 1.99)	−0.35 (−3.10, 2.48)	0.206
Sb	−0.69 (−1.86, 0.49)	Ref.	−2.33 (−4.47, −0.14) *	−2.53 (−4.81, −0.20) *	−1.99 (−4.53, 0.61)	0.206
Tl	−0.62 (−2.18, 0.96)	Ref.	−0.83(−3.07, 1.46)	0.63 (−2.01, 3.33)	−0.55 (−3.47, 2.45)	0.794
W	−0.97 (−1.88, −0.05) *	Ref.	−0.69 (−2.75, 1.41)	0.91 (−1.39, 3.25)	−1.72 (−4.20, 0.82)	0.342
U	−1.58 (−2.42, −0.74) **	Ref.	−2.19 (−4.19, −0.15) *	−2.46 (−4.61, −0.27) *	−3.74 (−6.03, −1.40) **	0.002

^a^ Adjusted for age, sex, race/ethnicity, BMI, annual family income, smoking, education, marital status, hypertension, diabetes, NHANES cycle, physical activity, serum cotinine, and urinary creatinine as well as other metals. * *p* < 0.05; ** *p* < 0.01; *** *p* < 0.001.

## Data Availability

The raw data utilized in this study are publicly accessible through the National Health and Nutrition Examination Survey at: https://www.cdc.gov/nchs/nhanes/index.htm (accessed on 10 November 2024).

## Supplementary Materials

The following supporting information can be downloaded at: https://www.mdpi.com/article/10.3390/toxics13040237/s1, Figure S1: Pairwise Pearson correlation matrix among ln-transformed urinary metals among study population in the NHANES 2007–2016; Table S1: Associations of single urinary metals with serum α-Klotho levels in the NHANES 2007–2016.
