# Supplementary material for: Independent and Combined Associations of Urinary Heavy Metal Exposures with Serum α-Klotho in Middle-Aged and Older Adults
Source: Toxics. 2025 Mar 24;13(4):237. doi: 10.3390/toxics13040237 (PMC12031166; doi:10.3390/toxics13040237)
Supplement: Supplementary file 1 [file toxics-13-00237-s001.zip › toxics-3519806-supplementary.pdf]

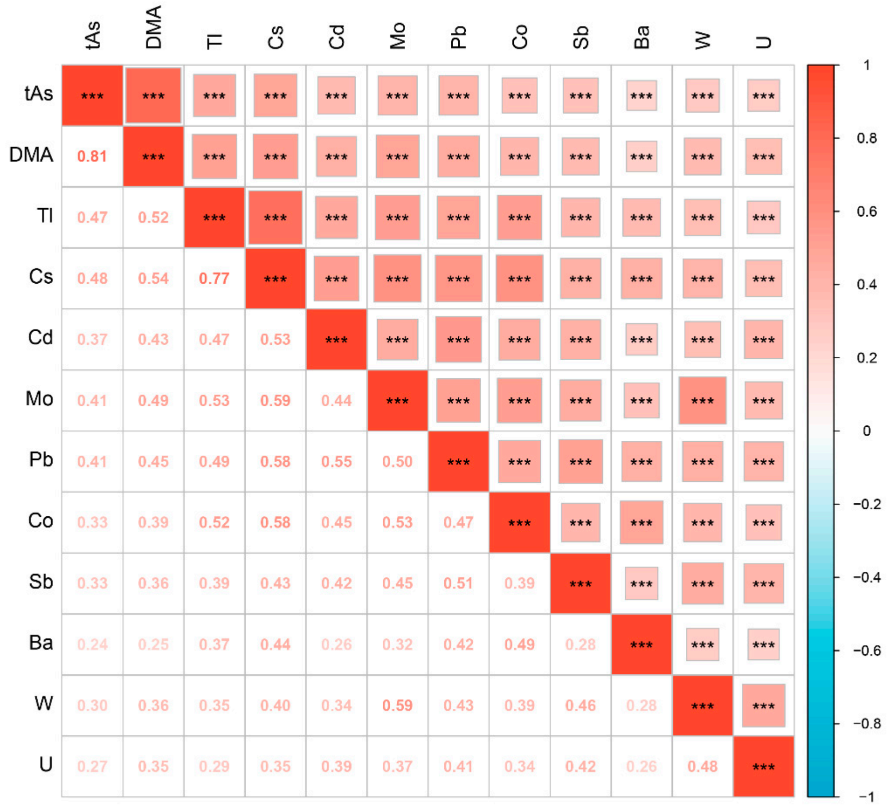

Figure S1. Pairwise Pearson correlation matrix among ln-transformed urinary metals among study population in the NHANES 2007-2016. Values and shading intensities represent Pearson correlation coefficients between two metals. ***, $p < 0.001$.

Table S1. Associations of single urinary metals with serum α-Klotho levels in the NHANES 2007-2016.

Metals	Percent change (95% CI) [a]					*p* for trend
	**Continuous**	**Q1**	**Q2**	**Q3**	**Q4**	
tAs	-0.23 (-0.92, 0.46)	Reference	-0.42 (-2.44, 1.65)	-1.31 (-3.42, 0.85)	-0.90 (-3.12, 1.38)	0.351
DMA	-0.16 (-1.18, 0.88)	Reference	-1.17 (-3.17, 0.86)	0.07 (-2.11, 2.30)	-0.83 (-3.18, 1.59)	0.792
Ba	**1.96 (1.19, 2.74)*** **	Reference	**4.30 (2.21, 6.47)*** **	**4.33 (2.05, 6.64)*** **	**5.12 (2.98, 7.30)*** **	**<0.001**
Cd	-0.42 (-1.40, 0.58)	Reference	0.35 (-1.68, 2.41)	-0.21 (-2.37, 2.00)	-0.21 (-2.74, 2.39)	0.781
Co	0.84 (-0.23, 1.91)	Reference	0.49 (-1.53, 2.54)	2.03 (-0.20, 4.32)	**2.95 (0.51, 5.46)** **	**0.010**
Cs	**2.67 (1.21, 4.14)*** **	Reference	1.26 (-0.79, 3.34)	**4.32 (1.98, 6.71)*** **	**4.39 (1.72, 7.14)** **	**<0.001**
Mo	**1.05 (0.05, 2.05)***	Reference	-0.28 (-2.28, 1.76)	1.12 (-1.09, 3.37)	0.70 (-1.75, 3.20)	0.400
Pb	0.13 (-0.89, 1.17)	Reference	0.98 (-1.06, 3.05)	0.63 (-1.56, 2.88)	0.70 (-1.82, 3.27)	0.670
Sb	-1.10 (-2.23, 0.03)	Reference	-1.85 (-3.93, 0.26)	-1.99 (-4.15, 0.22)	-2.06 (-4.45, 0.39)	0.128
Tl	0.98 (-0.25, 2.22)	Reference	-0.21 (-2.21, 1.83)	2.31 (-0.05, 4.73)	1.61 (-0.87, 4.15)	0.072
W	**-0.90 (-1.69, -0.10)***	Reference	-1.03 (-3.00, 0.98)	0.63 (-1.48, 2.78)	**-2.26 (-4.35, 0.13)***	0.161
U	**-1.65 (-2.43, -0.86)*** **	Reference	-1.79 (-3.73, 0.20)	**-1.99 (-3.78, -0.16)***	**-3.61 (-5.74, -1.42)** **	**0.002**

[a] Adjusted for age, sex, race/ethnicity, BMI, annual family income, smoking, education, marital status, hypertension, diabetes, NHANES survey cycle, physical activity, serum cotinine and urinary creatinine. * $p < 0.05$; ** $p < 0.01$; *** $p < 0.001$.
